# Supplementary material for: Fluorogenic, Subsingle-Turnover Monitoring of Enzymatic Reactions Involving NAD(P)H Provides a Generalized Platform for Directed Ultrahigh-Throughput Evolution of Biocatalysts in Microdroplets
Source: J Am Chem Soc. 2025 Mar 24;147(13):10903–15. doi: 10.1021/jacs.4c11804 (PMC11969528; doi:10.1021/jacs.4c11804)
Supplement: Supplementary file 1 — ja4c11804_si_001.pdf [file ja4c11804_si_001.pdf]

Supplementary Information

for

Fluorogenic, sub-single-turnover monitoring of enzymatic reactions involving NAD(P)H provides a generalised platform for directed ultrahigh throughput evolution of biocatalysts in microdroplets

Matthew Penner[1,#], Oskar James Klein[1,2,#], Maximilian Gantz[1,#], Friederike E. H. Nintzel[1], Anne-Cathrin Prowald[1], Sally Boss[2], Paul Barker[2], Paul Dupree[1], Florian Hollfelder[1,]*

Experimental Section

Synthesis

All NMR data were collected at 298 K using Bruker Avance spectrometers with ^{1}H resonance frequencies of 400 Hz. Chemical shifts (δ H) are reported in parts per million (ppm), to the nearest 0.01 ppm and are referenced to the residual non-deuterated solvent peak. Coupling constants (J) are reported in Hertz (Hz) to the nearest 0.1 Hz. Data are reported in the order: chemical shift (multiplicity, coupling constant(s), integration). For carbon NMR, chemical shifts (δ C) are quoted in ppm, to the nearest 0.1 ppm, and are referenced to the residual non-deuterated solvent peak. When present, DEPTSP135 peak phases are reported as p or n for positive (CH & CH_3) and negative (CH_2) respectively. Multiplicity is reported as: s = singlet; d = doublet; t = triplet; q = quartet; m = multiplet; or as a combination of these.

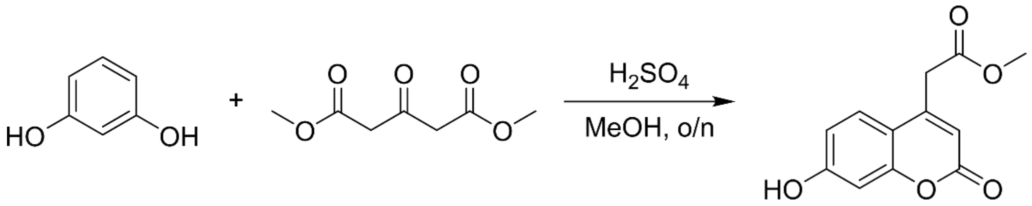

7-hydroxycoumarinyl-4-acetic acid methyl ester (I)

Concentrated sulphuric acid (45 mL) was added slowly to an ice-cooled solution of resorcinol (5.0 g, 45 mmol, 1 eq.) and dimethyl-1,3-acetonedicarboxylate (6.6 mL, 45 mmol, 1eq.) in methanol (70 mL). The reaction mixture was stirred at room temperature overnight, after which it was poured into ice-water (~ 500 mL) inducing precipitation. The product was filtered, washed with ice-cold water and then recrystallised from methanol to yield 7-hydroxycoumarin-4-acetic acid methyl ester as colourless, fibrous crystals (7.8 g, 33.3 mmol, 74% yield).

^{1}H NMR (400.03 MHz, DMSO-d^6) δ: 10.62 (b, 1H), 7.52 (d, J = 8.7 Hz, 1H), 6.80 (dd, J = 8.7, 2.3 Hz, 1H), 6.74 (d, J = 2.3 Hz, 1H), 6.24 (s, 1H), 3.95 (s, 2H), 3.65 (s, 3H)

^{13}C NMR (100.61 MHz, DMSO-d^6) δ: 170.1 (s), 161.8 (s), 160.6 (s), 155.5 (s), 150.0 (s), 127.2 (s, p), 113.5 (s, p), 112.6 (s, p), 111.7 (s), 102.8 (s, p), 52.7 (s, p), 37.1 (s, n)

HRMS [ESI(+)]: calculated for $C_{12}H_{11}O_5^+$ [M+H$^+$] 235.0601; found 235.0610 (+3.8 ppm)

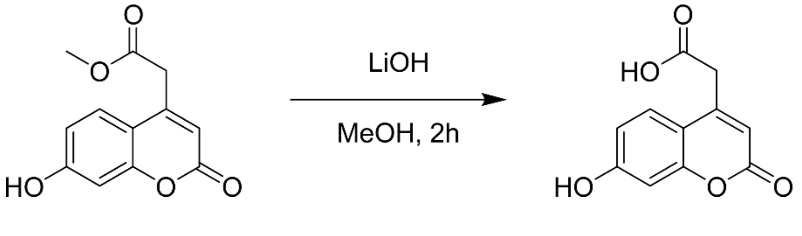

7-hydroxycoumarinyl-4-acetic acid (2)

Lithium hydroxide monohydrate (316 mg, 7.5 mmol, 2 eq.) was added to a mixture of 7-hydroxycoumarin-4-acetic acid methyl ester (883 mg, 3,8 mmol, 1eq) in water (20 mL) and THF (10 mL). After stirring overnight, the pH of the reaction mixture was set to ~2 with hydrochloric acid, inducing precipitation. The product was filtered, washed with ice-cold, dilute hydrochloric acid (~20 mL) and DCM (~10 mL) and dried to yield 7-hydroxycoumarin-4-acetic acid as a beige powder (558 mg, 2.5 mmol, 66% yield).

^{1}H NMR (400.03 MHz, DMSO-d^6) δ: 12.78 (b, 1H), 10.58 (b, 1H), 7.54 (d, J = 8.7 Hz, 1H), 6.81 (dd, J = 8.7, 2.4 Hz, 1H), 6.74 (d, J = 2.3 Hz, 1H), 6.23 (s, 1H), 3.83 (s, 2H)

^{13}C NMR (100.61 MHz, DMSO-d^6) δ: 170.7 (s), 161.2 (s), 160.2 (s), 155.0 (s), 150.2 (s), 126.8 (s, p), 113.0 (s, p), 112.0 (s, p), 111.4 (s), 102.3 (s, p), 37.3 (s, n)

HRMS [ESI(+)]: calculated for $C_{11}H_9O_5^+$ [M+H$^+$] 221.0445; found 221.0454 (+4.3 ppm)

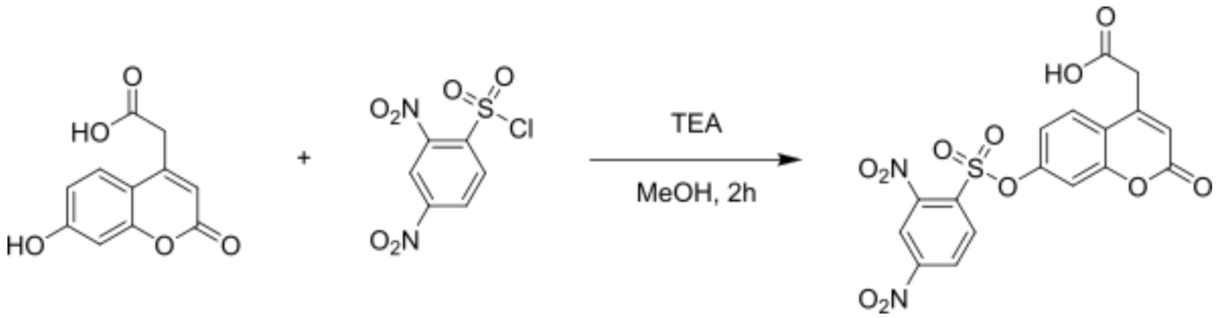

7-((2,4-dinitrophenyl)sulfonyl)oxycoumarinyl-4-acetic acid (1)

Under a flow of N_2, triethylamine (691 µL, 5.0 mmol, 2 eq.) was added to 7-hydroxycoumarinyl-4-acetic acid (558 mg, 2.5 mmol, 1eq.) in methanol (25 mL) and the solution stirred at room temperature for 10 minutes before cooling in an ice bath and portion-wise addition of 2,4-dinitrobenzenesulfonyl chloride. The reaction mixture was stirred for 2 hours in an ice-bath and under N_2, after which the solvent was removed *in vacuo*. The residue was taken up in water and the solution acidified to pH ~2 using hydrochloric acid, inducing precipitation. The product was filtered, washed with ice-cold, dilute hydrochloric acid (~10 mL) and DCM (~10 mL) and dried to yield 7-((2,4-dinitrophenyl)sulfonyl)oxycoumarinyl-4-acetic acid as a yellow/orange solid (851 mg, 1.9 mmol, 76% yield). Note (i): Higher purity could be achieved by dissolving the product in DCM and washing with dilute hydrochloric acid. However, the limited solubility of the product in DCM requires large amount of solvent to be used. Note (ii): Initially, the same condensation reaction was attempted using 7-hydroxycoumarinyl-4-acetic acid methyl ester (**I**) and found to be significantly less efficient.

^{1}H NMR (400.03 MHz, DMSO-d^6) δ: 12.90 (b, 1H), 9.13 (d, J = 2.2 Hz, 1H), 8.62 (dd, J = 8.7, 2.3 Hz, 1H), 8.36 (d, J = 8.7 Hz, 1H), 7.81 (d, J = 8.8 Hz, 1H), 7.38 (d, J = 2.4 Hz, 1H), 7.26 (dd, J = 8.8, 2.4 Hz, 1H), 6.58 (s, 1H), 3.94 (s, 2H)

^{13}C NMR (100.61 MHz, DMSO-d^6) δ: 170.4 (s), 159.0 (s), 153.7 (s), 151.6 (s), 149.9 (s), 149.2 (s), 148.1 (s), 133.6 (s, p), 130.7 (s), 127.7 (s, p)*, 121.3 (s, p). 118.9 (s),

118.2 (s, p), 116.9 (s, p), 110.6 (s, p), 37.0 (s, n) *This peak contains two carbon signals that overlap, both bonded to one proton, as confirmed *via* HSQC experiment.

HRMS [ESI(+)]: calculated for $C_{17}H_{11}N_2O_{11}S^+$ [M+H$^+$] 451.0078; found 451.082 (+0.9 ppm)

Excitation spectra and pK_a determination of SAFRAN product fluorophore 2

Fluorophore 2 was diluted to 100 µM in the following buffers: acetate (100 mM, pH 2-6), Tris-HCl (100 mM, pH 7-9), NaOH (100 mM, pH 10-12), and emission at 460 nm recorded using a Varioskan Lux plate-reader (Thermo Fisher) and a black 96-well plate (Nunc) in a total volume of 100 µl. To calculate the pK_a of fluorophore **2,** emission at 460 nm after exciting at 380 nm was plotted. The midpoint of the fitted sigmoid was taken as the pK_a of the fluorophore.

Protocols for small-molecule quantification

All fluorescence measurements were taken with a Varioskan Lux plate-reader (Thermo Fisher) or a Tecan Infinite 200 Pro (TECAN), measuring emission at 460 nm following excitation at 380 nm. Assay for reduced glutathione was performed using 90 µL of analytical solution containing Safran (50 µM) and 200 mM Tris-HCl (pH 7.0). To this, dilutions of reduced L-glutathione (Sigma) were added to make the total solution up to 100 µl per well. The reaction was incubated in plates, in the dark for 90 minutes.

Assay for NADH was carried out in the same manner, but with the addition of 52 µg/mL glutathione reductase from Baker's yeast (Sigma) along with 200 µM oxidised L-glutathione (Sigma) to the analytical solution. Dilutions of NADH (Carbolution) in 200 mM Tris-HCl (pH 7.0) were then added and the reaction was incubated in plates, in the dark for 90 minutes.

Assay for glucuronic acid in the picomole regime was carried out using 200 mM Tris-HCl (pH 8.0) supplemented with 40 µM of oxidised L-glutathione, 20 µM of NAD^+ (Sigma), 52 µg/mL glutathione reductase and 40 µM of Safran, with the addition of 10 µg/mL uronic acid dehydrogenase (Megazyme). 90 µL of this detection solution was added to dilutions of glucuronic acid (Sigma).

The gene-string for GH115 was received as a kind gift from Emma Master, codon-optimized for E. coli, and cloned tag-free into the pRSF expression vector. The inactive mutant E176A was cloned using SLIM, as previously described[1]. Assay for GH115 activity in *E. coli* lysate in 96-well format was carried out by first inducing cells expressing GH115 or the control enzyme *Sr*IRED grown to O.D. 0.8 in LB with 0.5 mM IPTG. After 12 h incubation at 16 °C, the cells were diluted to O.D. 1.0 and 10 µL of cells were added to 90 µL of analytical solution in 200 mM Tris-HCl (pH 7.0) supplemented with BugBuster (Merck), 500 µM Safran, 500 µM oxidised L-glutathione, 52 µg/mL glutathione reductase and 10 µg/mL uronic acid dehydrogenase.

Assay for NADPH was carried out using 90 µL of analytical solution containing 220 µM SAFRAN, 110 µM oxidized L-glutathione (Sigma), 1 µL/mL glutathione reductase from Baker's yeast (Sigma) and 200 mM Tris-HCl (pH 8.0). To this, dilutions of NADPH (Carbolution) in 200mM Tris-HCl (pH 8.0) yielding final concentrations from 0 to 100

µM, were added to make the total solution 100 µL per well. The fluorescence intensity was determined after 20 min and 2h incubation at room temperature.

Inoculation of a LB-Kan culture in deep-well plates (Greiner) from a glycerol stock of IRED X46 containing BL21 (DE3) cells (NEB) and beta lactamase respectively. The sequence for this construct is found below. The plate was incubated overnight (37 °C, 750 rpm). 30 µl of the culture were diluted in 920 µL of fresh LB-Kan media in a fresh deep-well plate and grown for 2h (37 °C, 750 rpm). Protein expression was induced with 0.2 mM IPTG, and the plate was incubated overnight (20 °C, 750 rpm). Cells were harvested by centrifugation (30 min, 4000g), the supernatant was removed, and cells were resuspended in 200 µl lysis buffer (100 mM Tris-HCl (pH 8.0), 4 mg/mL egg white lysozyme, 0.5 mM EDTA, 1 mg/mL polymyxin B) per well. The cell lysate was diluted 500-fold in 100 mM Tris-HCl (pH 7.0) and mixed in a 1:1 ratio with the substrate solution yielding a final concentration of 200 µM NADPH, 20 mM cyclohexanone and 40 mM cyclopropylamine. The solution was incubated at room temperature in Nunc 96-well plates (Thermo) for 0, 10, 30 and 60 min, respectively. For back-titration of unconverted NADPH, the SAFRAN reaction cascade was added in a 1:2 volume ratio, to a final concentration of 200 µM Safran, 100 µM oxidized L-Glutathione, 1 µL/mL Glutathione reductase from Baker's yeast (Sigma). Fluorescence was measured after 2h of incubation in the dark.
Limits of detection were defined as the lowest analyte concentration that can be stated to be statistically greater than the baseline measurement. In all cases, we use a one-tailed Welch's t-test, accounting for differences in variance between the baseline and analyte samples. The standard deviations of each group were used to calculate the test statistics, and the p-value was computed using python's scipy.stats module. Calibration curves were carried out using dilutions of Coumarin **2** to relate RFU to product concentration using identical buffer and plate reader conditions to the target experiment.

Chip design and microfluidic device fabrication

PDMS chip devices were prepared with the help of CAD software (DraftSight, Dassault Systems); both design files can be downloaded from our open access repository DropBase (http://openwetware.org/wiki/DropBase). The devices were fabricated as previously described[2]: briefly, following soft lithography fabrication[3,4], the PDMS chips were bonded to glass slides, and the channel surfaces modified to be hydrophobic using trichloro(1H,1H,2H,2H-perfluorooctyl)silane[5] (Sigma).

Quantification of droplet leakage

Water-in-oil droplets were produced at approximately 5 kHz using a double flow-focusing device with three inlets (height: 30 µm; width: 30 µm at the flow-focusing junction; **Figure S14**). Using syringe pumps (Nemesys), gas-tight syringes (SGE or Hamilton) and fine pore PTFE tubing (internal diameter 0.5 mm, external diameter 1.0 mm, Sigma), the two aqueous phases were mixed at the first junction. This stream was then pinched off by lateral oil (HFE-7500, fluorosurfactant-008 1%) flow from the third inlet, resulting in emulsion droplets with 20 pL volume. Droplet-making was monitored using an inverted microscope (SP981, Brunell Microscopes), with a high-speed camera (Miro eX4, Phantom Research). For the experiment in described in 3A, the first aqueous phase consisted of 300 µM Coumarin **2** dissolved in Tris-HCl (200 mM, pH 7.0), while the second phase consisted of the buffer only. Droplets were

collected as described previously[2], in an inverted microcentrifuge tube filled with HFE-7500, with the modification that 1% fluorosurfactant-008 was used. After 10 minutes of droplet collection, the first (coumarin-containing) aqueous inlet was removed and replaced with a plug, and the flow rates were again adjusted so that 20 pL droplets were being generated, containing Tris-HCl (200 mM, pH 7.0) only. These were collected into the same collection vessel for a further ten minutes. Following this, the collection chamber was inverted several times and incubated for 24 h. 10 µL of droplets were then transferred to the stage of an EVOS FL microscope and imaged using the DAPI channel.

The same experiment was then carried out by first generating droplets containing first 25 µM and then 50 µM coumarin product, and performing the same inversion and incubation at 23 °C. At intervals of two days, five days and sixteen days, the droplets were passed through a FADS detector, and the population fluorescence measured. Each time, this resulted in two separate distributions, which were modelled as gaussian. After defining the midpoint of the gaussian functions as 1.0, the two distributions were plotted and overlayed.

Determining limits of detection in droplets

20 pL water-in-oil droplets were generated in the manner described above using a double flow-focusing device with three inlets (Figure S14, https://openwetware.org/wiki/DropBase:droplet_generation_3_inlets). The oil inlet (HFE-7500, fluorosurfactant-008 1%), was used to create droplets with a mixed aqueous phase deriving from the two other inlets. One of these inlets used Tris-HCl (200 mM, pH 8.0) and other used the same buffer supplemented with 500 nM fluorophore **2**. The outlet of this flow-focusing device was connected to the inlet of the FADS detector, and the droplet traces were recorded and analysed using a custom LabView script to extract the singlet droplet peaks on-the-fly. In this case with the chosen flow rates, the singlet width was identified as 310 – 450 microseconds, representing the timeframe over which the droplet causes a deflection of signal voltage from the baseline measured by the photomultiplier tube and detector. The peak voltage was identified and recorded for each of these events. These data were recorded and stored as histograms of droplet counts per voltage bin, and Gaussians fit to the data to extract the mean and standard deviation of the population. Goodness-of-fit was also determined for each Gaussian and is reported in the relevant figure legend. The relative flow rates between the aqueous inlets were adjusted to systematically vary the concentration of fluorophore **2** in the measured droplets, ensuring that measurements were measured under consistent conditions between populations. The fitted means and standard deviations were then plotted as a function of fluorophore concentration, and the limit of detection reported as the lowest concentration of fluorophore that had a statistically higher signal than the 0 nM fluorophore **2** population, confirmed using Welch's t-test. The details of the applied t-test are found in the appropriate figure legend.

The *limit of detection* is the concentration where the signal is statistically different from the background of an empty droplet – and we take this as our assay resolution. Our claim of sensitivity of 30 nM is based on this, regardless of background. This value is compared to the enzyme concentration (estimated as 1,000,000 molecules per droplet[6], 83 nM), suggesting that we can detect fewer product molecules than there

is enzyme present. Based on these considerations we conclude that the resolution of the assay allows distinction of a sub-single turnover signal on top of the background deriving from turnover of GSH under these assumed conditions. For practical applications, when applying droplet selections, the ability to oversample each phenotype is important when attempting to detect trace activities. This is because each phenotype will display a distribution around its true value, which will only be resolvable if the median of the distribution is above the assay resolution – 30 nM of product formed. By oversampling, droplet assays can therefore leverage these distributions to infer even these trace phenotypes around the level of noise.

Quantification of uronate dehydrogenase activity

30 pl droplets were generated as above using two phases. The first phase was the analyte phase, containing 1 mM glucuronic acid (Megazyme), dissolved in Tris-HCl (200 mM, pH 7.0). The second phase was the detection phase. This was comprised of 500 µM of oxidised L-glutathione, 20 µM of NAD^+ (Sigma), 52 µg/mL glutathione reductase and 500 µM of Safran, with the addition of 10 µg/mL uronic acid dehydrogenase (Megazyme), dissolved in Tris-HCl (200 mM, pH 7.0). The negative control droplets replaced the first phase with Tris-HCl (200 mM, pH 7.0). After generating negative control droplets and collecting as described earlier, droplets containing the mixed glucuronic acid and detection solution were collected into the same chamber. The chamber was inverted, and immediately connected to a FADS device, where RFU data of the mixed population were collected and subsequently plotted. This was repeated at 40 minutes and after overnight incubation.

Enrichment of GH115 using fluorescence activated cell sorting

Water-in-oil droplets were produced as described above, using the same double flow-focusing device with three inlets. The first aqueous flows contained a 1:1000 mixture of the two glycosidase E. *coli* lines WT:E176A. To prepare this model library, the two lines were grown separately to OD 0.6 in LB-Kan at 37 °C, and then induced separately for 16 h at 16 °C using 0.5 mM IPTG. This ensures that cells are synchronised in stationary phase by the time they are screened – an important consideration to minimise background phenotypic variation[7]. The cultures were matched to O.D. 1.0, and then mixed 1:1000 WT:E176A. The cells were then diluted to an OD of 0.04 in LB-Kan and drawn up into the tubing before injection on-chip, to prevent cell settling to the bottom of the syringe.

The second aqueous flow contained lysis agent – 1 µL/mL rLysozyme (Merck, 71110-3) – and assay reagents. This mixture contained 0.05% w/v beechwood xylan (Megazyme), 10 µg/mL uronate dehydrogenase (Megazyme), 1 mM NAD^+ (Sigma), 5 mM oxidised L-glutathione (Sigma), 52 µg/mL glutathione reductase from Baker's yeast (Sigma) and 1 mM Safran in 100 mM Tris-HCl (pH 7.0). Note that concentrations given are concentrations in the syringe, and not in the droplet, where the 1:1 aqueous flow rate used diluted the concentration two-fold. Droplets were collected as described above. The droplets were incubated for one hour at 23 °C to permit cell lysis and catalysis.

Using a syringe pump and syringes containing HFE-7500 with fluorosurfactant-008 (1%), droplets were re-injected onto the sorting device at a rate of 800 Hz. As droplets passed a 90° bend, they were illuminated with a 375 nm laser, passed through optical fibres (Thorlabs), and light emitted at 90° was collected using an optical fibre (Thorlabs)

connected to a 380 nm low-pass filter and a photomultiplier tube (H8249, Hamamatsu Photonics). This amplified signal was processed using a data acquisition card at 38 kHz (National Instruments). A peak detection algorithm was used as previously described[8], which calculated the fluorescence intensity and peak width (LabView 8.2, National Instrument). Sorting was triggered based on gating of this population, as described previously[9] and events were manually inspected to ensure only the singlet population was being selected.

After collection in a 1.5 mL microcentrifuge tube designed for low DNA retention (DNA LoBind, Eppendorf), the droplets were de-emulsified by the addition of 200 µL 1H,1H,2H,2H-perfluorooctanol (97%, Alfa Aesar). 100 µL 2 ng/µL salmon sperm DNA (Invitrogen) was then added, and the mixture vortexed and centrifuged at 1000 x g for one minute. The aqueous (upper) layer was removed and transferred to a fresh microcentrifuge tube. Contaminants were then removed and the DNA was concentrated, using a DNA clean and concentrate kit (Zymo) according to the manufacturer's instructions. DNA was eluted in 7 µL H_2O, and 1 µL was used to transform 25 µL high competency E. cloni (E.cloni 10G ELITE, Cambridge bioscience), according to the manufacturer's instructions.

Following selection on a LB-kanamycin plate, approximately 500 colonies were re-suspended in LB-Kan, and the plasmid DNA extracted using a miniprep kit (GeneJET). This DNA was re-transformed into *E. coli* BL21(DE3) (NEB), according to the manufacturer's instructions. Following selection and growth on a LB-kanamycin plate, 90 colonies were picked and transferred to a deep-well 96-well plate. Each well contained 500 µL LB-Kan, and three wells were selected as negative controls (inoculated with BL21-E176A_GH115), and three wells as positive controls (BL21-WT_GH115). The cultures were shaken and grown to saturation overnight at 37 ˚C. 10 µL of this culture was then used to inoculate another deep-well 96-well plate containing 490 µL LB-Kan, and this plate was shaken and grown for two hours. At this point, when the cultures reached approximately O.D. 0.8, they were induced using IPTG (0.5 mM) and vigorously shaken at 16 ˚C for 12 h. The cultures were then centrifuged for 20 minutes at 4000 x g, and the supernatant removed. 200 µL of a cell lysis master mix (10x BugBuster (Merck) in 100 mM Tris-HCl, pH 7.0) was used to resuspend the cultures, which were then vigorously shaken for 30 minutes at 16 ˚C. The resulting lysate was then centrifuged for 20 minutes at 4000 x g, and the supernatant transferred to a clean 96-well plate. A second 96-well plate was then prepared, with each well containing 90 µL of 0.05% w/v beechwood xylan, 10 µg/mL uronate dehydrogenase and 5 mM NAD^+ in 100 mM Tris-HCl (pH 7.0). 10 µL of the soluble lysate was added to each well, and the reaction progress monitored using absorbance at 340 nm.

Enrichment of SrIRED using fluorescence activated cell sorting

5 mL LB-Kan culture were inoculated with a 1mL OD 600 1.0 glycerol stock of chemically competent BL21(DE3) cells (NEB) carrying the IRED plasmid and the pRSF (negative control) plasmid respectively and grown to an OD of 0.8 (37 °C, 300 rpm). Protein expression was induced with 0.2 mM IPTG and the cultures were incubated overnight (20°C, 220 rpm). The two cultures were combined to yield a cell suspension of 2% IRED X46 with 98% empty vector cells. Then, the suspension was diluted in 100mM Tris-HCl (pH 7.0) and 20% Percoll (Sigma) to a final OD 600 of 0.067.

Droplets were generated at 4000 Hz using a flow-focusing device with the cell solution (as described above) and substrate solution (10 µL/mL BugBuster, 1mM EDTA, 200 µM NADPH (Carbolution), 20 mM cyclohexanone (Fluka), 40 mM cyclopropylamine (from pH-adjusted stock of 500 mM cyclopropylamine (Sigma) in 100 mM Tris-HCl pH 7.0) and HFE (Fluorochem) with 2% RAN surfactant). Flow rates were adjusted to yield droplets with an approximate volume of 15 pL and an average cell occupancy of 0.25. Droplets were collected as described above.
After incubation for 1.5 h, picoinjection with 15 pL was performed at 500 Hz (1.5 V constant sine wave) with 400 µM Safran, 2 µM Coumarin 2, 200 µM oxidized L-Glutathione, 2 µL/mL Glutathione reductase from Baker's yeast (Sigma) in a pico-injection device. FADS was performed using the same custom-made sorting device (fabricated as previously described) as described above with the exception that 2% Ran was used. Droplets were collected in a DNA LoBind tube (Eppendorf). The plasmid DNA was recovered as described above. The eluate of 6 µL was transformed into 50 µL high competency E. cloni (E.cloni 10G ELITE, Cambridge Bioscience), as described above. Following selection on a LB-kanamycin plate, 12 colonies were resuspended in LB-Kan, the plasmid DNA extracted using a miniprep kit (GeneJET) and sent for Sanger sequencing.

Directed evolution

Library generation was performed using error-prone PCR according to the manufacturer's instructions (library size: 4 million colony-forming units post-transformation; mutations per gene 6 ± 5). The library was transferred from 10 G to BL21, then subject to induction with 0.5 mM IPTG over 12 h at 22 °C and dilution to OD 0.02. This was then encapsulated with 0.05% w/v BWX, alongside coupling reagents. The cell lysis and reaction occurred in droplets for one hour before the sorting procedure was started – the top 1000-most fluorescent events were sorted from 3.6 million droplets processed, with droplets processed at an average rate of 1.1 kHz over a window of approximately one hour. The DNA from the sorted droplets was recovered by re-transformation into ultra-competent *E. coli* and then transferred back to the BL21 screening host. These BL21 colonies were then randomly selected for lysate screening and divided into wells of a 96-well plate, using the catalytically inactive E176A and WT enzyme as negative and positive controls respectively. This process was performed in duplicate. Following growth stage normalization and overnight induction at 20 °C with IPTG, lysate was tested for hydrolase activity against 0.5% w/v beechwood xylan. This process was also followed for the input library, using randomly picked BL21 from the unsorted library as inputs. 39 wells from the output were identified as having higher than average wild-type activity, while only four members from the input had high-than-input activity. Five members with higher-then-wildtype activity were selected and subjected to Sanger sequencing.

Purification of engineered variants

Mutants identified in selections were cloned into a His-tagged construct using SLIM to introduce the required mutations, as described previously[1]. *AxyAgu*115A was then purified as described previously[10], with several modifications. BL21 (DE3) cells transformed with the pRSF-GH115 constructs were grown in pre-culture to saturation overnight at 37 ˚C (5 mL LB-Kan). The main culture was then inoculated with this preculture (LB-Kan, 1 L), and grown to OD 0.4-0.8 shaking at 37 °C. The cultures were

then induced with IPTG (0.5 mM) and left to shake for 16 h at 26 °C . The cultures were then centrifuged (6000 x g, 20 minutes), and the supernatant removed. Each pellet was resuspended in binding and lysis buffer (30 mL, NaCl 300 mM, HEPES 50 mM, glycerol 5%, imidazole 5%, pH 7.0). The cells were subject to sonication (80 %, 15 min, 10 s on/5 s off). The lysed cells were then centrifuged at 17500 xg for 20 minutes, and the supernatant transferred to 2 mL Ni-NTA resin (Qiagen). This resin had been pre-equilibrated in wash buffer (NaCl 300 mM, HEPES 50 mM, glycerol 5%, imidazole 50 mM, pH 7.0). The mixture was left on a roller at 4 ^{o}C for 2 h. The mixture was then subject to gravity column filtration and washed three times with 5 mL wash buffer. The protein was then subject to elution with 2 washes of 2.5 mL elution buffer (NaCl 300 mM, HEPES 50 mM, glycerol 5%, imidazole 250 mM, pH 7.0). The eluted protein was then concentrated and subject to buffer exchange in a size-selection filter (Amicon 30K) and was exchanged into storage buffer (NaCl 300 mM, HEPES 50 mM, pH 7.0). The concentrated protein was snap-frozen in liquid nitrogen and stored at -80 °C until further use. Protein purity was confirmed by SDS-PAGE.

Kinetic characterization of engineered variants

Concentrated protein was thawed on ice from -80 °C. A detection mixture was then constructed, comprising 10 µg/mL uronate dehydrogenase and 1 mM NAD^+ in 200 mM Tris-HCl (pH 7.8). Engineered GH115 was then added to the detection mixture to a final concentration of 1 µg/ul, as measured by protein A280 (nanodrop). 50 µl of this mixture was then mixed with 50 µl of reaction mixture comprising diluted BWX in 200 mM Tris-HCl (pH 7.8) and the absorbance at 340 nm measured using a plate reader (Spectramax iD5, Molecular Devices). Maximum reaction rates were calculated from these curves, and normalized by subsequent Bradford assay protein concentrations[11] to yield the specific activity of each GH115 variant at each concentration of beechwood xylan. This was performed in triplicate and used to derive the Michaelis-Menten parameters. The effective concentration of the (4OMe)-GlcA epitope used was calculated according to the manufacture's specifications that 13% of xylose residues per chain were substituted with glucuronic acid.

Gene sequence (WT *AxyAgu*115A):

ATGGACTTTACCCTGAACCAAGAAATGCTGATGACGGACACCAAATCGGGCGCA
CTGTTTTACCAAGAAGAAGAAGCACTGAGCGGCGTTCGCAAAATTGCCAATAAA
GTCATGCATGATGTCGAACTGGTGTTTGGCTACCAGCCGGAAGCTACCAAAGAT
CGTGACATGCTGTCGCGCCATGCGGTTCTGTATGGCACGGTCGGTCACAGCCC
GCTGCTGGATGAACTGAACAAGAAAGAACTGATCGACCTGACCGAAATTGCGG
GTAAACGCGAAGTGTTTCTGTTCCAGGTGGTTGATCAGCCGATTCAAGGCGTGG
AAAAAGCACTGGTTATCGCTGGTAGCGACAAACGTGGCACCATTTACGGTCTGT
TTCATCTGAGCGAAAAACTGGGCGTCTCTCCGCTGGTTGATTGGAGTGGTGTTC
TGCCGGCCCGCAAAGAATCATTCTCGCTGAAAGGCGACTATAAATACGTGTCAA
AAGAACCGTCGGTTAAATACCGTGGCTTTTTCATCAACGATGAATGGCCGGCCTT
CGGTAACTGGTCCGCAAAAAATTTTGGCGGTTTCAACGCAGAAATGTACGATCA
CGTGTTTGAACTGCTGCTGCGCCTGAAGGGTAACTATCTGTGGCCGGCGATGT
GGTCAGCCCGTTTCAATGATGACGGCCCGGGTCTGGCTAACGTTGAACTGGCG
GATGAATATGGCGTCATCATGGGTGCATCGCATCACGAACCGTGCCTGCGTTAC
GGCGAAGAATATAAATACCTGCGCGGCCCGGATAGCATTTATGGTGACGCGTGG

AACTTTATCACCAATCGTGAAGGTATTACGAAATTCTGGGAAGATGGCCTGAAAC
GCACCGGTCATTTTGAAAATATTATCACGATCGGCATGCGTGGTGAAGCTGATAC
CAAAATTATGGGCGAAGATGCGACGCTGGAAGACAACATCAATCTGCTGCGCGA
TGTGATTCAGACCCAAAACAAACTGATCAAAGAACACGTGAACCCGAATCTGAA
AGAAGTTCCGCGTATGCTGGCGCTGTACAAAGAAGTGGAACCGTTTTTCTATGG
CGATGAAAATACCCCGGGTCTGATCAACTCCGAAGAACTGGAAGACGTTATTCT
GATGCTGTGTGATGACAACCATGGCAATCTGCGTACCCTGCCGACGGAAGATAT
GCGCAAACACTCCGGCGGTTACGGCATGTATTACCATTTTGACTATCACGGCGG
TCCGGTGTCATACGAATGGATTAATAGCTCTTATCTGCCGAAAATCTGGGAACAG
ATGACCATGGCTTATGATTTCGGCGTTCGTGACCTGTGGATCGTGAACGTTGGT
GATATTGCCACGCAGGAACTGCCGCTGAGCTTTTTCCTGGACCTGGCCTACGAT
TTTGACAAATGGGGCACCAACGCAATCAATAAAACCGATGACTATACGAAACAGT
GGATTGAACAGCAATTTGCCGGCGTGTTCAATCTGGAACAAAAAGATAAAGTTTT
CGAACTGCTGAACGGTTATACCAAAATCGCCCATAATCGTCGCCCGGAAGCAAT
GAACGTCGATGTGTATCATCCGGTGAATTACCACGAAACCGACCAGCTGCTGGA
TCGCATTGACCATCTGCTGGGTCTGGCGGAAGAACTGTACCAAGAAGTTGATCA
GCAACACTTTACCGCGTATTTCGCCCTGGTCTATTACCCGACGGTGGGCAACCT
GAATCTGCAGAAAATGTGGCTGCTGAACGGTAAAAATAAATATGCGGCCCAACTG
AATCTGATTGAAGCAAACAAACTGGCTGAACAGGTGAAAGCCTGCCTGAAACGT
GATCAAGAAATTGTTGACGAATACCATACCATCGCAGATGGCAAATTTTATGGCAT
GGGTCTGAGCGAACATATCGGTTTCGTCCACTGGAATGAAGATGAAAACAAAAA
TCCGGTGCTGTCTTATGTTCTGCCGGTCAACAAACCGCGTCTGCTGGTGTCTAT
TGATGGCACCGAACTGCGCAGTGAAGGTTCCCCGTGGCACGTCAATACGCTGC
CGCTGGTTGATTTTCTGGAACCGGACGTCAACCAGGCCTCCTTCACCATCAGTT
CCGTGTCAGAAAAGAAAGCGGAATATCATATTTCTACGGATCAAGACTGGCTGAG
CTGTTCTGCAGCTAATGGCGTTCTGGATGGTAAAAACAAACTGAGTGAAACCATC
CACGTTTTTGTCGATCGTGACGGCCTGGCAGATCAGGCTGAAGGTCGCATCAC
CGTGAAAACGCCGGTGGGCAAAGTTACCATTGTCGTGCCGGTTGTCAACAATGA
TTTTACGAATTACCCGGATATGACCTTCGTTGACACGAAAGGCTATATTTCGATCG
AAGCGGAACATTTTGCCACCCAGAAAGCAACGGAAAATCTGGATGGTACCCTGA
ACCGCTTCGAAGTGCTGGACGGCTATGGTAAAACGCTGAGCGCGATTAAAGCCT
TTCCGACCGATACGCATTACCAAGTGGGCAAAGACGCTCCGTTTGTTGAATATCA
CTTCGTCACCCAGGAAGCGGGTGTTTATGAACTGGAATTTTACCTGCAACCGTC
TAATCCGGTTACCCGTGAAGGCACGATGTACGCGGGTATCCAGGTCAACGAAAA
TGATGTGGACGTTATTAACGTCCTGCCGGATGGTTATCATGTGGACGGCCCGCA
CTGGGGTATTGATGTGATCAACAATATTCGCACCACGAAAACCAAAATCACGTGC
GAACAGGGCCTGAACAAACTGCGTATTTACGCTGTTAGTCCGGGTTTTGCGCTG
GAAAAAATTGTCATCTATCCGGATGGCAAAAAACTGGCTAACTCCTACCTGGGTC
CGAATGAAACCTACTATGTGGGTCGTTAA

Table S1: Survey of directed evolution studies for glycosidase activity that have reported kinetic parameters of the evolved enzymes. Note the difference in assessing the success of the directed evolution campaigns by calculating the increases brought about by the acquired mutations by using the substrate used in the assay (column 5) and the actual desired target reaction (columns 6).

Target substrate	Substrate used in evolution campaign	Target enzyme	Screening platform	Maximum fold-increase (for the **substrate** used in evolution assay; $\boldsymbol{k_{cat}/K_M}$ or other **chosen success metric** as indicated)	Maximum fold-increase (target substrate)	Ref.
**Cellobiose and lactose**	fluorescein di-glucopyranoside	*Cs* β-gluco-sidase	FACS, double emulsions	*N.d.* *(1.8-fold on model substrate pNPGlu)*	1.13 (Cellobiose) 1.7 (Lactose)	[24]
**A-antigen**	MU-Type1A$_{penta}$	Sp3GH98	Microtitre plates	170 (in k_{cat}/K_M)	15-25% increased removal of Type 1A/1A-Le^b antigen[a]	[25]
**β-glycosides**	cellobiose, X-Glu, and 4-β-MUG mixture	β-glycosi-dase	Agar plates (growth)	100 (in whole-cell turnover number)	*N.d.*[b]	[26]
**Cellobiose**	Cellobiose	*Aa*BGL1	Agar plates (growth)	1.05 (in k_{cat}/K_M)	1.05 k_{cat}/K_M[c]	[27]
**7-β-Xylosyltaxane**	PNP-Xyl	Lxyl-p1–2	96-well plates	1.6 (as specific activity of lysate)	1.47 k_{cat}/K_M	[28]
**1,4-linked beta glucans**	AZO-CMC	CelStrep	Agar plates (colori-metric)	3.3 (as specific activity of lysate)	30% improved sacchari-fication of pretreated *A. donax* biomass	[29]

[a] Measured by antigen staining with immunofluorescence. Bait substrate was conjugated to MU fluorophore, compared to target substrate conjugated to red blood cells.
[b] No enzyme kinetics comparing unevolved with evolved mutants were reported.
[c] The identified mutation was found to improve the product inhibition of the enzyme.

Table S2: Survey of works using FADS and a comparison of their parameterization

Lit.	Fluorophore	Target	Laser (nm)	Stage or optical fibres?	Sorting frequency (Hz)	Droplet size (pL)	Incubation time
Neun et al 2023[12]	Fluorescein	Glycosyl hydrolase	488	Stage	> 1000	4	2 days
Schnetler et al[13]	Fluorescein	Phosphotri-esterase	488	Stage	500-3000	3	30 mins – 3 hours
Schnetler et al[14]	Fluorescein	Phosphodi-esterase	488	Stage	800	3	Not Reported
Holstein et al 2021[2]	BODIPY	Protease	488	Stage	200	30-45	3 days
Kintses et al 2012[15]	Fluorescein	Sulfatase	488	Stage	1000	15	1 hour
This work	Coumarin 2	Glycosyl hydrolase + Imine reductase	375	Optical fibres	800	30	1 day

Table S3: Assumptions underlying estimates of assay sensitivity in droplets .

Parameter	Quantification	Reference
Droplet volume	**20 pL**	*This work*
E. coli volume	**0.7 μm^3**	[16]
Volume dilution between cell and droplet	**28570 x**	
Assay resolution:<br>• concentration	**30 nM**	*This work, S10*
• number of molecules<br>\	**361,000**	
Glutathione concentration in cell	**6.6 mM**	[7]
Glutathione in droplet:<br>• concentration	**230 nM**	
• number of molecules	**2,800,000**	
Enzyme per cell (and per droplet, after lysis)<br>number of enzyme molecules<br>concentration	**1,000,000**<br>**83 nM**	[6]

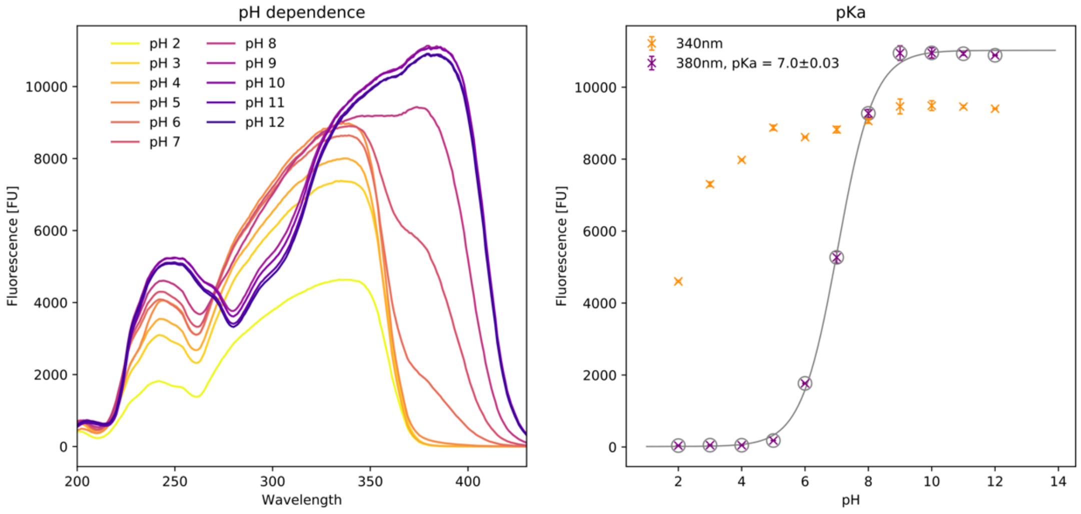

Figure S1: Excitation, Emission and pK_a of fluorophore 2
Fluorophore 2 (100 µM) was excited at various wavelengths in buffers of various pH (acetate (100 mM, pH 2-6), Tris-HCl (100 mM, pH 7-9), NaOH (100 mM, pH 10-12)), and emission at 460 nm recorded. Emission at 460 nm after exciting at 380 nm was plotted and used to calculate the pK_a of fluorophore **2**, using the midpoint of the fitted sigmoid.

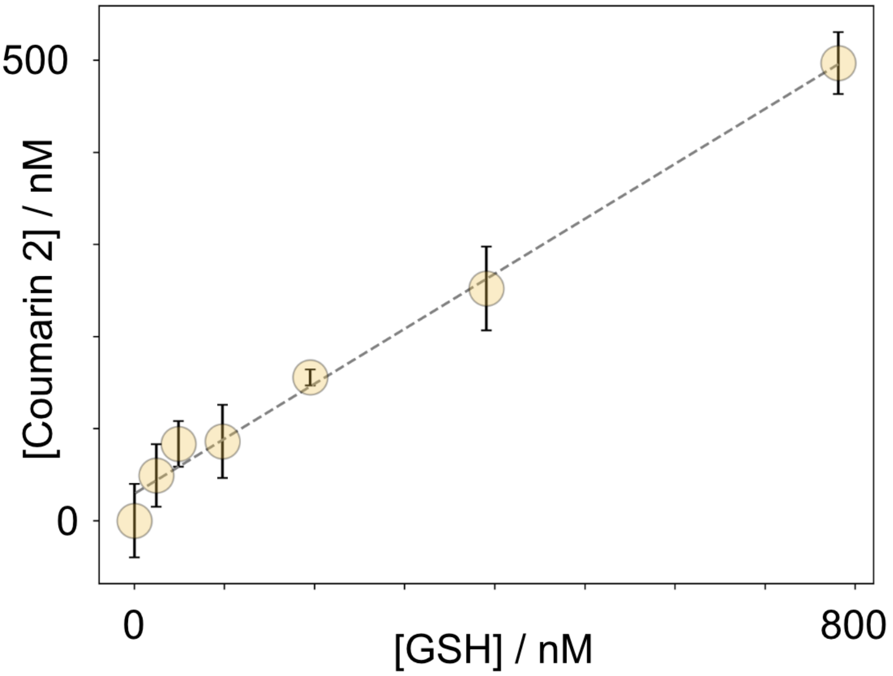

Figure S2: Detection of nanomolar GSH using SAFRAN
The nM range of the GSH-fluorescence response signal. Each reaction was performed in triplicate at 23 °C, in 200 mM Tris-HCl (pH 7.0), excited at 380 nm and its emission measured at 460 nm. Fluorescence signal was quantified after 90 minutes of reaction in the dark. The limit of detection was 50 nM, as defined by a signal significantly above baseline ($p = 0.04$, Welsh's one-sided t-test, $n = 3$ per sample)

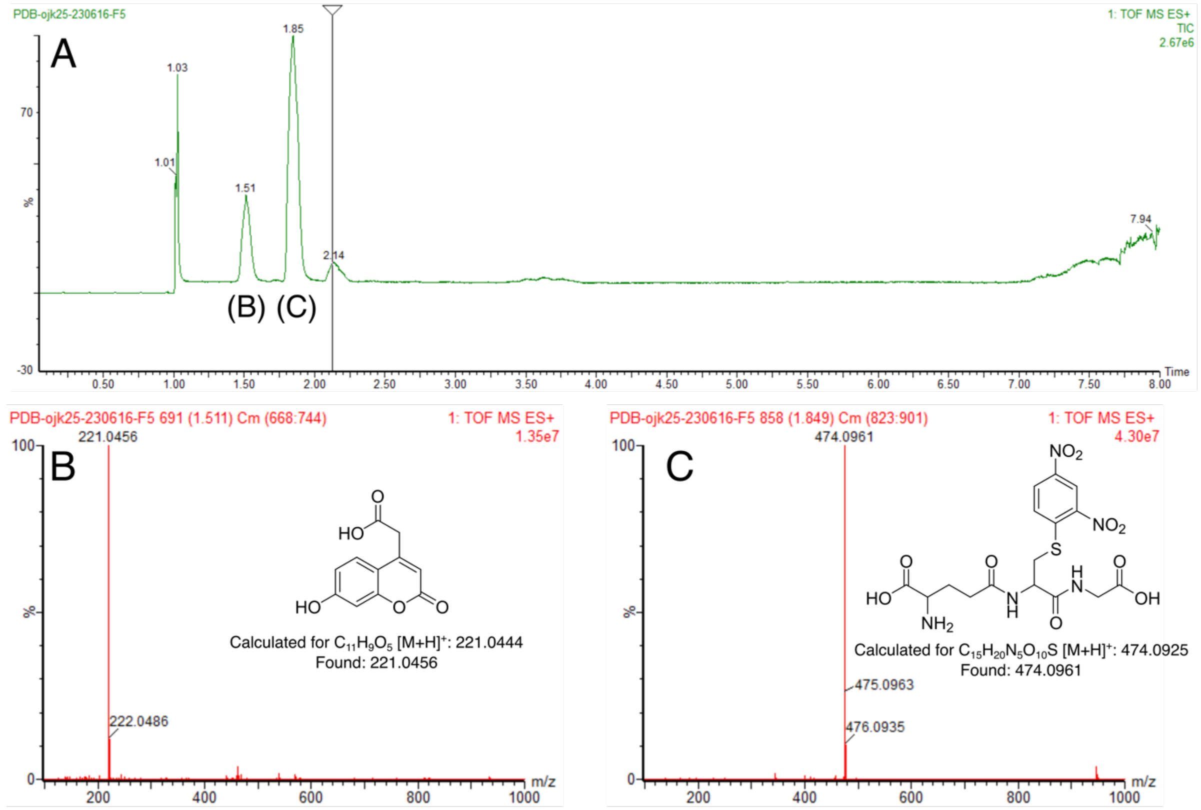

Figure S3: Mass spectrometry of reaction products.
LC-MS chromatogram of the reaction of 100 μM Safran and 100 μM reduced glutathione, in Tris-HCl (100 mM, pH 7.0) at 23 °C. (A). Mass spectra of the individual peaks show full conversion to the free fluorophore **2** (B) and dinitrobenzyl-modified glutathione (C).

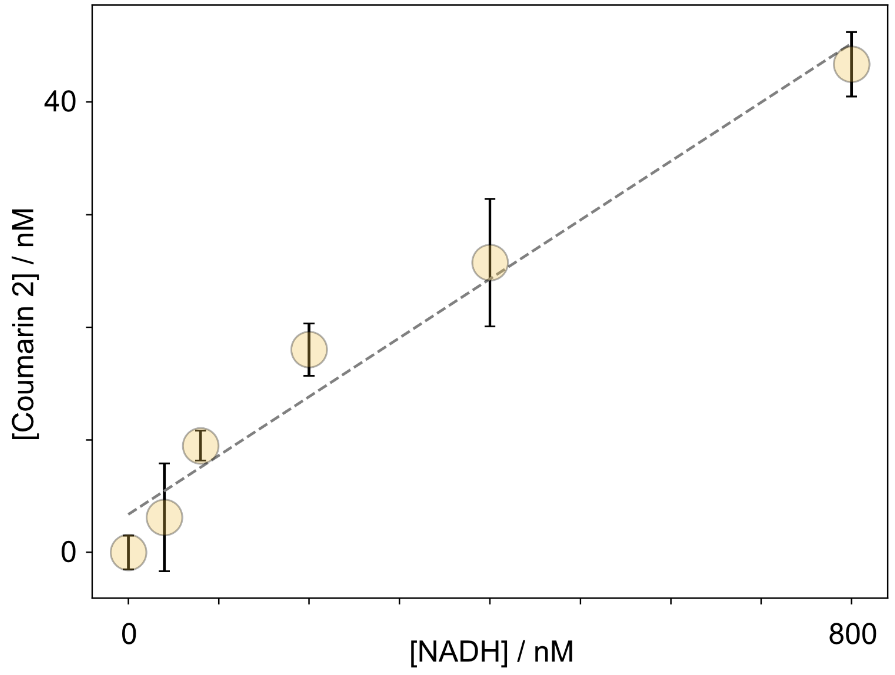

Figure S4: Oxidation of nanomolar [NADH] to NAD^+ with concurrent reduction of glutathione, monitored by SAFRAN conversion to fluorophore 2
The smallest concentrations tested that was statistically distinctly greater than the baseline found by Welsh's t-test, was 25 nM (with a p-value of 0.001, n = 3 per sample). Therefore, the limit of quantification in plates of the NADH-assay was defined as 50 nM NADH. Reactions were performed in Tris-HCl (pH 7.0), with 1.2 mM GSSG and 2.6 µg glutathione reductase per 100 µL well. 500 µM SAFRAN was used, and reactions proceeded for 1 h at 23 °C before measurement of fluorescence emission at 460 nm following excitation at 360 nm.

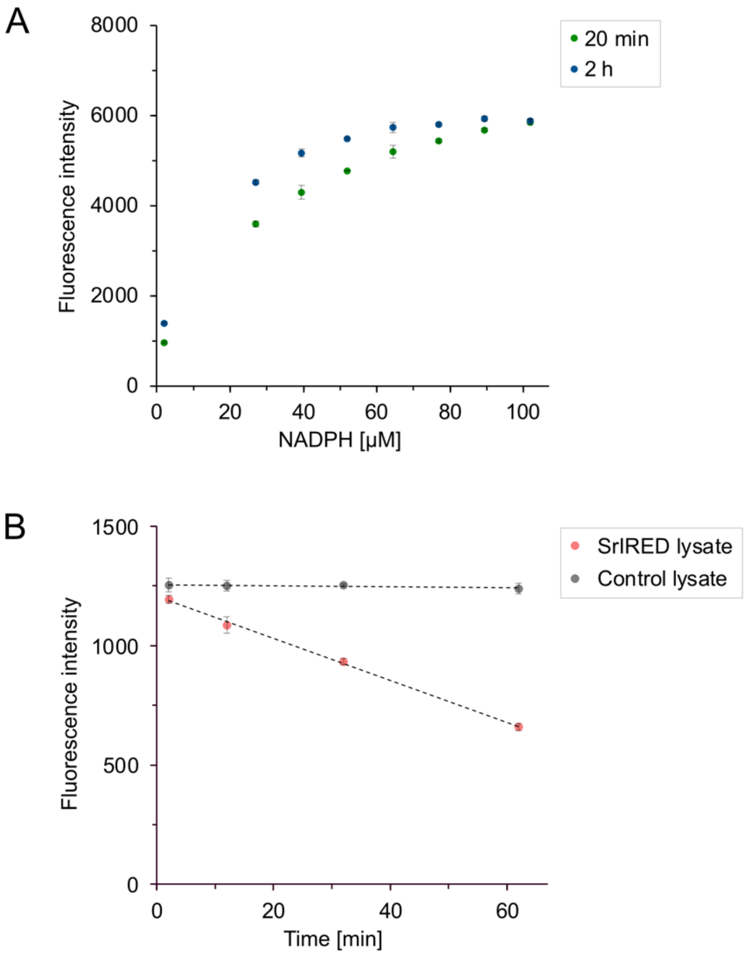

Figure S5: Oxidation of nanomolar [NADPH] to $NADP^+$ with concurrent reduction of glutathione, monitored by Safran conversion to fluorophore 2. Here we establish SAFRAN for IREDs in the reverse direction to sense NADPH depletion in plates.
(A) Correlation between NADPH concentration and fluorescence signal up to 100 µM, and signal stability over 2 h. NADPH was prepared in increasing concentrations in a 96-well plate and the SAFRAN cascade was added. Fluorescence intensity was measured after 20 min and 2 h in a spectrophotometer. While linearity between NADPH concentration and fluorescence intensity was only observed at low concentrations of NADPH, increase in fluorescence intensity was detected up to 100 µM. The signal was stable over 2h incubation time, however at the costs of decreased dynamic range at high NADPH concentrations.
(B) Signal decrease over time when applying the reverse SAFRAN cascade with IRED lysate. Clarified and diluted cell lysate of *Sr*IRED expressing E. Coli BL21 and control E. Coli BL21 was incubated with 100 µM NADPH,10 mM cyclohexanone and 20 mM cyclopropylamine in

100 mM Tris-HCl buffer pH 7. After 0 min, 10 min, 30 min and 60 min, the reaction was quenched and analysed by addition of the SAFRAN cascade. For the *Sr*IRED lysate, fluorescence intensity over time reported on a linear rate of NADPH depletion during the IRED reaction. For the control lysate, no decrease in fluorescence signal was observed, indicating for the absence of any background NADPH consuming reaction.

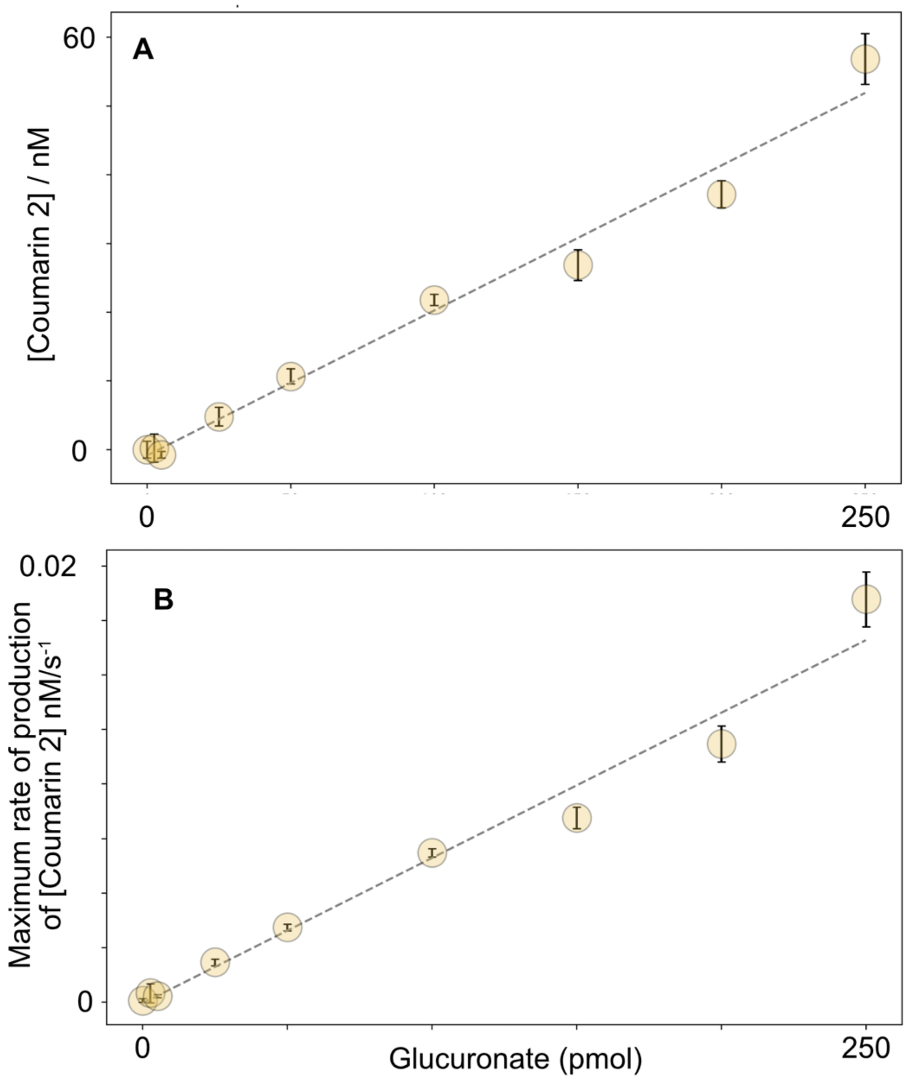

Figure S6: Quantification of monosaccharides in the picomole regimes
(A) Relation of maximum [Coumarin 2] and GlcA added. The entire cascade reaction (Figure 1) was allowed to occur for one hour, with monitoring emission at 460 nm when exciting at 380 nm. Maximum emission was used to linearly quantify the glucuronic acid present. 1 mM GSSG and 1 mM NAD^+ were used. **(B)** Relation of maximum rate of [Coumarin 2] increase and glucoronate added.

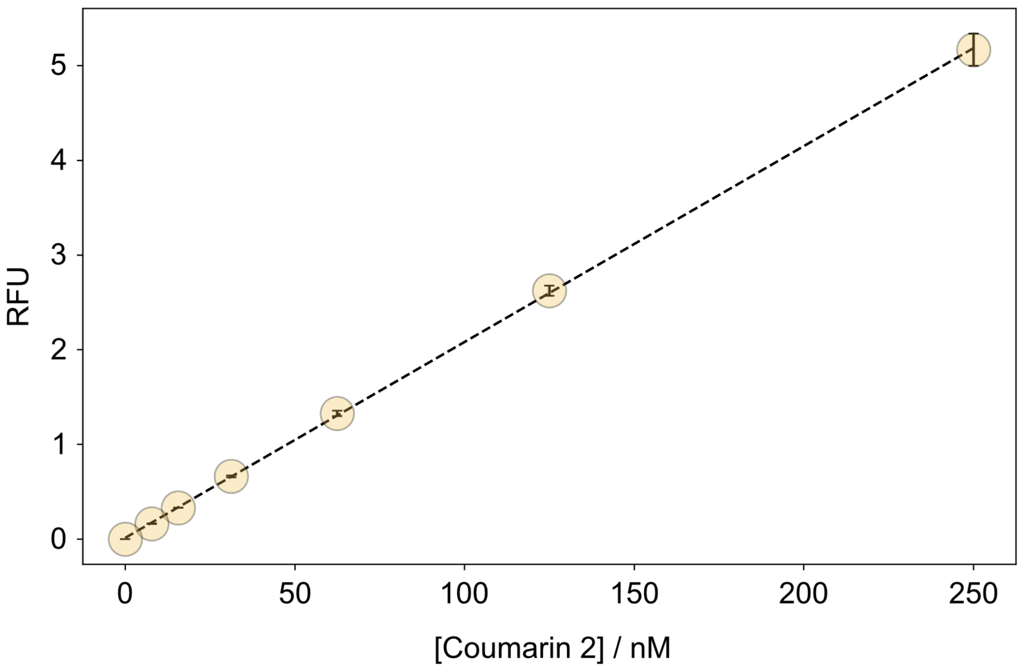

Figure S7: Coumarin 2 dilutions in *E. coli* lysate demonstrating signal linearity and limits of detection

Microtiter plate experiments diluting coumarin 2 in working concentrations of *E. coli* lysate, in Tris-HCl (100 mM, pH 8.0). Each condition was carried out in triplicate. Error bars represent the standard deviation of the value. Where error bars are too small to be seen, the standard deviation is low. The limit of detection was found to be 7.8 nM by comparing the baseline to the lowest measured concentration of coumarin 2 ($p = 0.0001$, Welsh's one-sided t-test, $n = 3$ per sample).

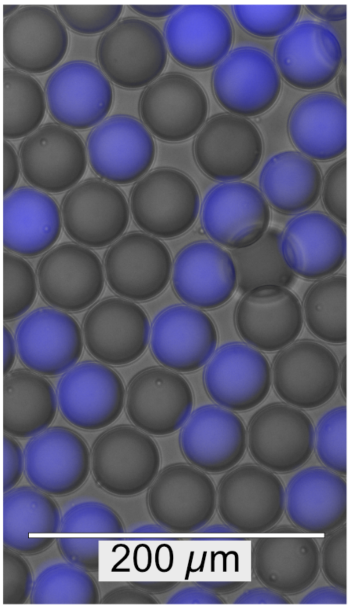

Figure S8: Fluorescence microscopy demonstrating fluorophore compartmentalization after overnight incubation
Droplets containing only Tris-HCl (200 mM, pH 7.0) or supplemented with coumarin **2** (100 μM) were generated separately, and then mixed in an incubation chamber, with overnight incubation at 23 ^{o}C. They were then applied to the stage of an inverted fluorescence microscope (EVOS FL), imaged and visualized with an overlay of the brightfield and DAPI channels.

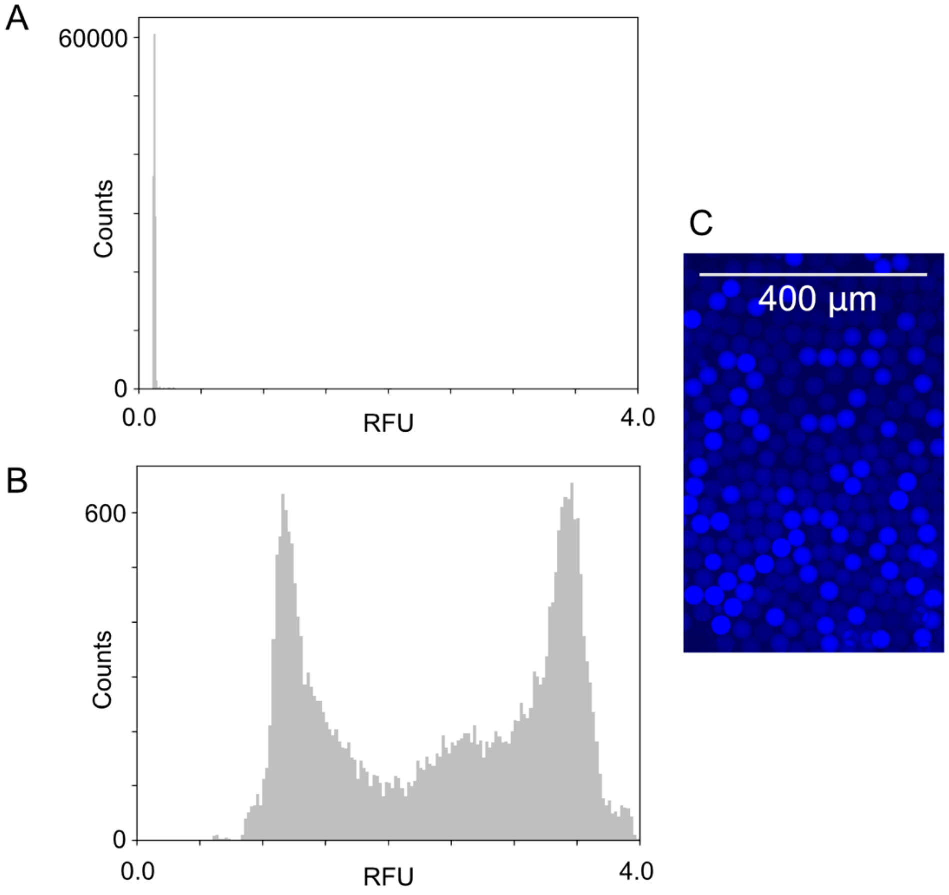

Figure S9: Droplet incubations of the glucuronic acid detection cascade using SAFRAN.

Droplet fluorescence signals from 30 pl emulsions to demonstrate that the cascade reagents react quickly to signal, and that the entire cascade is compatible with the droplet microenvironment. Droplets containing cascade reagents supplemented with 500 µM glucuronic acid and droplets containing only cascade reagents were generated and mixed in the same incubation vessel. These were incubated and their population fluorescence measured after a **(A)** 10 minute incubation and **(B)** one-day incubation. **(C)** Fluorescence microscopy images of the population after a one-day incubation.

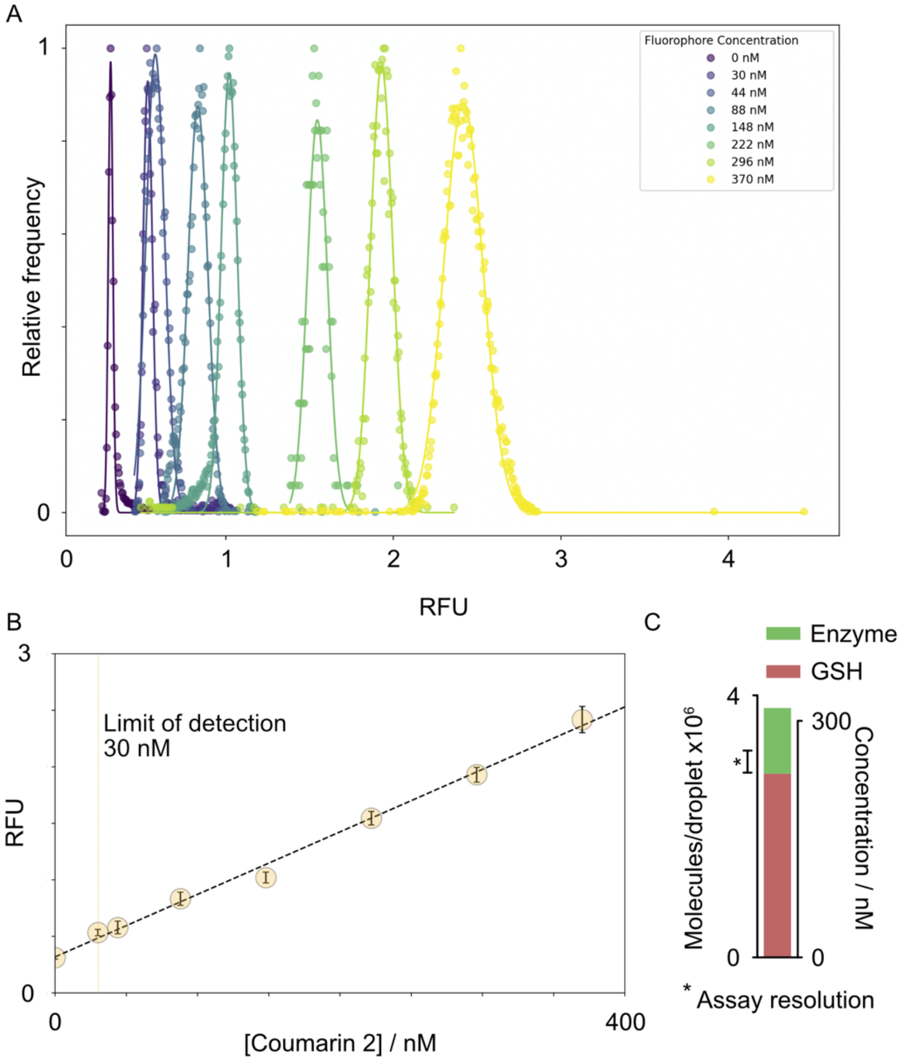

Figure S10: Droplet populations containing titrated coumarin 2 on-chip
(A) Fluorescence measurements of droplets obtained in our FADS rig (described in Methods). In separate experiments droplet populations with increasing concentrations of fluorophore **2** were generated and flowed past the detector to measure the fluorescence in each droplet. The data were then overlayed in this figure to visualise the relationship of concentration and fluorescence. In each experiment, the counts in each RFU bin have been normalized by the most populated bin. Gaussian fits are shown for each population. The R^2 goodness of the Gaussian fit are (in ascending order of coumarin **2** concentration): 0.98 0.97,0.97,0.96,0.99,0.85, 0.97 and 0.98. **(B)** Mean and standard deviation of the RFU of each droplet population, where error bars represent one standard deviation. The limit of detection of 30 nM was established using the empty droplets (n = 28203) and the 30 nM droplets (n = 2001). A one-sided Welsh's t-test using these data yielded a negligible ($<10^{-5}$) p-value between the 0 nM and 30 nM droplets. **(C)** Comparison of determined resolution/limit of detection to concentrations of relevant cellular analytes – intracellular GSH and overexpressed enzyme. Concentrations are given assuming one cell in a 20 pL droplet, and raw values are derived from table S3. The assay resolution of 30 nM is shown for scale as a starred bar.

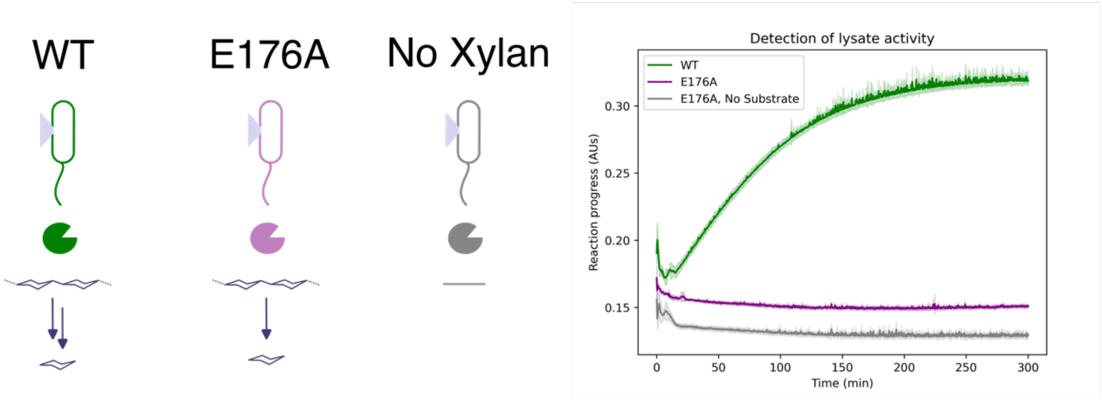

Figure S11: E176A confers low *AxyAgu*115A activity in *E. coli* lysate
The active site mutant E176A reduces activity of the enzyme *AxyAgu*115A in *E. coli* lysate to below the limit of product quantification, as measured in plates. Glucuronic acid quantification using NADH absorbance was carried out when induced lysate was applied to 0.5 mg/mL beechwood xylan in plate assays, with each condition in triplicate.

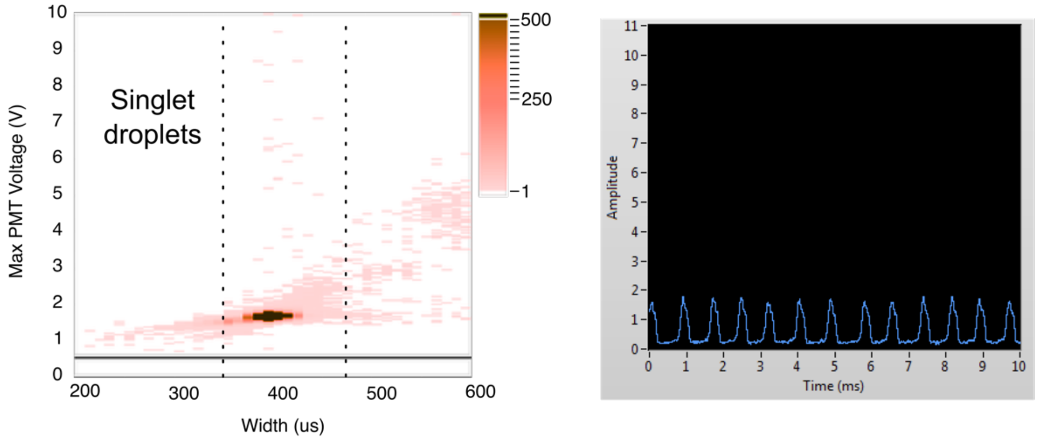

Population statistics

Droplets analyzed	275628
Average Rate	804 Hz
Droplets sorted	393
Fraction sorted	0.14%

Figure S12: Voltage trace from droplet enrichment
Peak voltage trace of all deviations from the baseline detected and recorded as droplet events, without size-gating for peak width. Highlighted counts represent peaks identified that satisfy expected singlet droplet sizes. Also pictured is a raw voltage-time trace showing droplet events, showing the droplets have a constant width upon recording, and background is consistent, allowing for accurate size-gating. Sort statistics are displayed below.

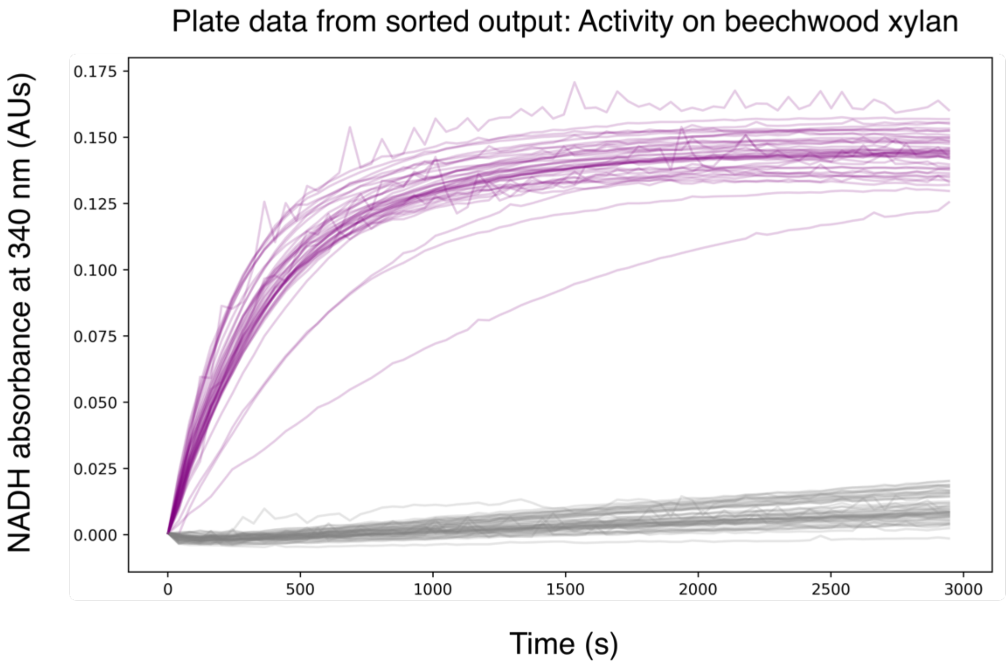

Figure S13: Re-screening of hits from droplet screening in plate format.
Lysate activity of induced BL21 transformed with the sorted library output, measured in 96-well plates, with induced lysate applied to beechwood xylan, with the coupling enzyme uronate dehydrogenase, and 1 mM NAD^+ (T = 21˚C). Reaction progress is monitored using absorbance of NADH at 340 nm. Clones counted as positive were highlighted in purple, while negative clones were highlighted in grey. These traces include three positive controls (found in the main population of positive clones) and two negative controls (found in the main population of negative clones). One of the original three negative controls was lost due to cellular debris interfering with the absorbance signal and was omitted from this plot. Using these data, the true positive rate of sorting was calculated to be 43%.

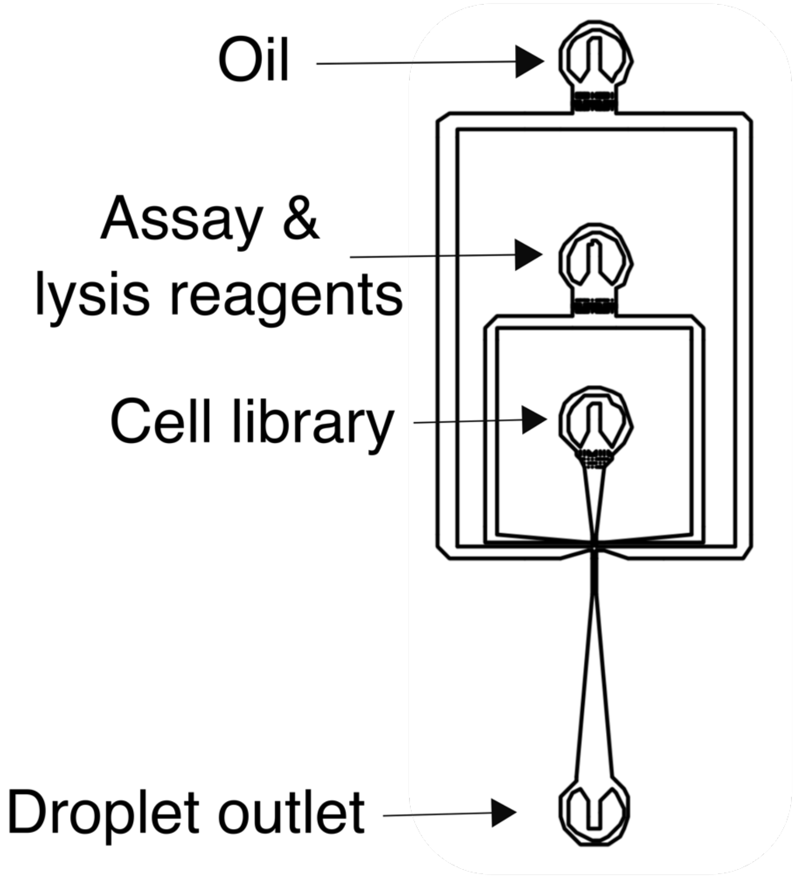

Figure S14: Double-input flow-focusing chip for droplet generation
The exact dimensions and a downloadable design file for this chip can be found on DropBase:
https://openwetware.org/wiki/DropBase:droplet_generation_3_inlets
The same can be found for the FADS device here:
https://openwetware.org/wiki/DropBase:droplet_electrosorting_6

Supplementary Note 1: Calculation of enrichment score for glycosidases

The method of Baret et al[17] was used to define enrichment. This value η is defined as:

$$\eta = \frac{N_{+}^{Sort}}{N_{-}^{Sort}} \Big/ \frac{N_{+}^{Input}}{N_{-}^{Input}}$$

Where $N_{+}^{Sort}$is the number of hits in the sorted population, N_{-}^{Sort} is the number of negatives in the sorted population, N_{+}^{Input} is the number of hits in the input library, and $N_{-}^{Input}$is the number of negatives in the input library. Based on our data, where 39/90 of the output were positive, and 1/1000 of the input were positive, we obtain η = 765.

Using the method of Zinchenko et al[18], the degree of enrichment is quantified using the same variables but by the alternative equation:

$$\eta' = \frac{N_{+}^{Sort}}{N_{+}^{Sort} + N_{-}^{Sort}} \Big/ \frac{N_{+}^{Input}}{N_{+}^{Input} + N_{-}^{Input}}$$

Using the Zinchenko formula, the degree of enrichment is found to be η' = 433.

Supplementary Note 2: Calculation of enrichment score for imine reductases

Using the method of Baret et al, η = 98
Using the method of Zinchenko et al, η' = 33

Supplementary References

[1] J. Chiu, P. E. March, R. Lee, D. Tillett, *Nucleic Acids Res.* **2004**, *32*, e174.
[2] J. M. Holstein, C. Gylstorff, F. Hollfelder, *ACS Synth. Biol.* **2021**, *10*, 252–257.
[3] J. C. McDonald, D. C. Duffy, J. R. Anderson, D. T. Chiu, H. Wu, O. J. Schueller, G. M. Whitesides, *Electrophoresis* **2000**, *21*, 27–40.
[4] Y. Xia, G. M. Whitesides, *Angew. Chem. Int. Ed Engl.* **1998**, *37*, 550–575.
[5] M. Gantz, S. Neun, E. J. Medcalf, L. D. van Vliet, F. Hollfelder, *Chem. Rev.* **2023**, *123*, 5571–5611.
[6] F. Gielen, R. Hours, S. Emond, M. Fischlechner, U. Schell, F. Hollfelder, *Proc. Natl. Acad. Sci.* **2016**, *113*, E7383–E7389.
[7] P. Apontoweil, W. Berends, *Biochim. Biophys. Acta BBA - Gen. Subj.* **1975**, *399*, 1–9.
[8] B. van Loo, M. Heberlein, P. Mair, A. Zinchenko, J. Schüürmann, B. D. G. Eenink, J. M. Holstein, C. Dilkaute, J. Jose, F. Hollfelder, E. Bornberg-Bauer, *ACS Synth. Biol.* **2019**, *8*, 2690–2700.
[9] Napiorkowska, M, Kaminski, T, C. Hollfelder, F, *Prep.* **n.d.**
[10] R. Yan, T. V. Vuong, W. Wang, E. R. Master, *Enzyme Microb. Technol.* **2017**, *104*, 22–28.
[11] M. M. Bradford, *Anal. Biochem.* **1976**, *72*, 248–254.
[12] S. Neun, P. Brear, E. Campbell, T. Tryfona, K. El Omari, A. Wagner, P. Dupree, M. Hyvönen, F. Hollfelder, *Nat. Chem. Biol.* **2022**, *18*, 1096–1103.
[13] J. D. Schnettler, O. J. Klein, T. S. Kaminski, P.-Y. Colin, F. Hollfelder, *J. Am. Chem. Soc.* **2023**, *145*, 1083–1096.
[14] J. D. Schnettler, M. S. Wang, M. Gantz, H. A. Bunzel, C. Karas, F. Hollfelder, M. H. Hecht, *Nat. Chem.* **2024**, *16*, 1200–1208.
[15] B. Kintses, C. Hein, M. F. Mohamed, M. Fischlechner, F. Courtois, C. Lainé, F. Hollfelder, *Chem. Biol.* **2012**, *19*, 1001–1009.
[16] H. E. Kubitschek, *J. Bacteriol.* **1990**, *172*, 94–101.
[17] J.-C. Baret, O. J. Miller, V. Taly, M. Ryckelynck, A. El-Harrak, L. Frenz, C. Rick, M. L. Samuels, J. B. Hutchison, J. J. Agresti, D. R. Link, D. A. Weitz, A. D. Griffiths, *Lab. Chip* **2009**, *9*, 1850–1858.
[18] A. Zinchenko, S. R. A. Devenish, B. Kintses, P.-Y. Colin, M. Fischlechner, F. Hollfelder, *Anal. Chem.* **2014**, *86*, 2526–2533.
